# Nigerian Honey Ameliorates Hyperglycemia and Dyslipidemia in Alloxan-Induced Diabetic Rats

**DOI:** 10.3390/nu8030095

**Published:** 2016-02-24

**Authors:** Omotayo O. Erejuwa, Ndubuisi N. Nwobodo, Joseph L. Akpan, Ugochi A. Okorie, Chinonyelum T. Ezeonu, Basil C. Ezeokpo, Kenneth I. Nwadike, Erhirhie Erhiano, Mohd S. Abdul Wahab, Siti A. Sulaiman

**Affiliations:** 1 Department of Pharmacology and Therapeutics, Faculty of Medicine, Ebonyi State University, Abakaliki 480214, Ebonyi State, Nigeria; nnwobodo@yahoo.com (N.N.N.); etejoe2006@gmail.com (J.L.A.); ugoochi94@gmail.com (U.A.O.); 2 Department of Pediatrics, Faculty of Medicine, Ebonyi State University, Abakaliki 480214, Ebonyi State, Nigeria; ctezeonu@gmail.com; 3 Department of Internal Medicine, Faculty of Medicine, Ebonyi State University, Abakaliki 480214, Ebonyi State, Nigeria; ezeokpo_bc@yahoo.co.uk; 4 Department of Pharmacology and Therapeutics, College of Medicine, University of Nigeria, Enugu 400211, Enugu State, Nigeria; kenneth.nwadike@unn.edu.ng; 5 Department of Physiology, College of Health Sciences, Usmanu Danfodiyo University, Sokoto 840212, Sokoto State, Nigeria; erhianoefe@yahoo.com; 6 Department of Pharmacology, School of Medical Sciences, Universiti Sains Malaysia, Kubang Kerian 16150, Kelantan, Malaysia; msuhaimikb@usm.my (M.S.A.W.); sbsamrah@usm.my (S.A.S.)

**Keywords:** honey, diabetes mellitus, hyperglycemia, hyperlipidemia, dyslipidemia, lipid profile, alloxan, rats

## Abstract

Diabetic dyslipidemia contributes to an increased risk of cardiovascular disease. Hence, its treatment is necessary to reduce cardiovascular events. Honey reduces hyperglycemia and dyslipidemia. The reproducibility of these beneficial effects and their generalization to honey samples of other geographical parts of the world remain controversial. Currently, data are limited and findings are inconclusive especially with evidence showing honey increased glycosylated hemoglobin in diabetic patients. It was hypothesized that this deteriorating effect might be due to administered high doses. This study investigated if Nigerian honey could ameliorate hyperglycemia and hyperlipidemia. It also evaluated if high doses of honey could worsen glucose and lipid abnormalities. Honey (1.0, 2.0 or 3.0 g/kg) was administered to diabetic rats for three weeks. Honey (1.0 or 2.0 g/kg) significantly (*p* < 0.05) increased high density lipoprotein (HDL) cholesterol while it significantly (*p* < 0.05) reduced hyperglycemia, triglycerides (TGs), very low density lipoprotein (VLDL) cholesterol, non-HDL cholesterol, coronary risk index (CRI) and cardiovascular risk index (CVRI). In contrast, honey (3.0 g/kg) significantly (*p* < 0.05) reduced TGs and VLDL cholesterol. This study confirms the reproducibility of glucose lowering and hypolipidemic effects of honey using Nigerian honey. However, none of the doses deteriorated hyperglycemia and dyslipidemia.

## 1. Introduction

Diabetes mellitus is a metabolic disorder associated with an increased risk of cardiovascular disease (CVD), a main cause of mortality in diabetes [1]. Although several factors account for increased CVD risk in diabetes, abnormalities of lipid metabolism are important contributors [2]. Hence, in addition to controlling hyperglycemia, treatment of dyslipidemia is inevitable to reduce cardiovascular events in diabetes [3]. While the current agents employed for the treatment of dyslipidemia are effective, these drugs are not easily affordable to many patients [4]. Besides, the use of some of these agents is associated with undesirable side effects. Some of these factors compel patients to seek alternative and complementary medicines. Even though complementary and alternative medicines are easily accessible and more affordable, their use is not without drawback. These agents are of unproven efficacy, and there are great concerns for safety and risk of untoward adverse effects [5].

One such complementary medicine that has gained wide attention in the past decade is honey. The anecdotal use of honey dates back to 2100–2000 BC [6]. Research in the past few years has provided convincing evidence in support of antioxidant, antibacterial and the wound healing properties of honey [7,8,9]. With regard to other reported beneficial effects, especially metabolic and cardiovascular effects such as antihypertensive, hypolipidemic, hypoglycemic and antidiabetic effects of honey, data are limited and the findings remain inconclusive particularly in clinical studies. At the moment, due to paucity of data, it remains unclear if these reported beneficial metabolic effects of honey can be reproduced using any honey sample or is restricted to a specific honey or certain honeys. Evidence has revealed that there is variation in the composition of honey. This variation depends on certain factors including geographical origin and botanical sources of the nectar [10]. Other factors such as climate, environment and processing techniques also contribute to variation in honey composition [10,11]. The variation in honey composition may influence the pharmacological effects derived from the honey samples. This leads to another uncertainty as to whether findings obtained with a honey sample from a particular geographical origin or floral source can be generalized to honey samples from other geographical parts or botanical sources of the world [12].

More worrisome is the evidence from a study which showed that honey increased glycosylated hemoglobin in diabetic patients [13]. This finding appears to suggest a potential deteriorating effect of honey on glycemic control. However, it was later explained that this particular finding should not be generalized to all honey samples as a result of two factors. These factors are: the administered high doses and the high glucose content of the administered honey [14]. In that particular study, graded doses of honey were administered orally to diabetic patients for eight weeks. The initial dose (1.0 g/kg/day) was increased by 0.5 g/kg/day every two weeks till the end of the study. Besides, the honey in question had a considerably higher glucose content than that found in most honey samples [15]. It was suggested that these two factors would invariably enhance glycosylation and contribute to increased glycosylated hemoglobin in diabetic patients [14]. This potential deterioration of glycemic control resulting from honey administration may also aggravate dyslipidemia in diabetes. Therefore, this study was carried out to investigate if Nigerian honey could reduce hyperglycemia and ameliorate lipid abnormalities in alloxan-induced diabetic rats. It also aimed to evaluate if high doses of honey could deteriorate glucose and lipid derangements in alloxan-induced diabetic rats.

## 2. Materials and Methods

### 2.1. Chemicals

Alloxan and glucose were purchased from Sigma-Aldrich, MO, USA. All other reagents used were of analytical grade.

### 2.2. Animals

The Wistar rats were purchased from animal house unit, Nsukka, Nigeria. The animals were acclimatized for at least 2 weeks. Two rats were housed per cage and maintained in a well ventilated animal room with temperature of 25–27 °C and 12-h light/dark cycle. The rats had free access to rat chow and portable water *ad libitum*. The animals were handled with humane care and in accordance with institutional guidelines of Ethics Committee of Ebonyi State University (EBSU/UREC/15/FCM/004) and international guidelines on the Use and Handling of Experimental Animals [16].

### 2.3. Honey

Honey was purchased from a bee farm in Ebonyi State, Nigeria. The honey had a NAFDAC (National Agency for Food and Drug Administration Control) registered number. The honey was dissolved in drinking water and prepared freshly each time it was administered.

### 2.4. Induction of Diabetes Mellitus

Diabetes mellitus was induced in overnight fasted male Wistar rats (180–220 g) via intraperitoneal administration of 150 mg/kg body weight (BW) of alloxan dissolved in normal saline. Another group of fasted rats was administered normal saline without alloxan. The rats were given 20% (w/v) glucose solution to prevent fatal post-alloxan hypoglycemia. Forty-eight hours (48 h) post-alloxan administration, rats with elevated fasting blood glucose (BG) concentrations ≥250 mg/dL were considered diabetic and included in the study.

### 2.5. Treatment

The animals were randomly divided into five groups. All the groups (except group 5) consisted of 6 rats. Group 5 comprised 5 rats because a rat died few days to the end of the treatment period. Using oral canula, the rats were administered drinking water or honey once daily between 8:00 and 9:00 a.m. for 3 weeks as follows:

- Group 1: Non-diabetic rats administered 1 mL/kg BW of drinking water
- Group 2: Diabetic rats administered 1 mL/kg BW of drinking water
- Group 3: Diabetic rats treated with 1.0 g/kg BW of honey
- Group 4: Diabetic rats treated with 2.0 g/kg BW of honey
- Group 5: Diabetic rats treated with 3.0 g/kg BW of honey

Before the commencement of treatment, body weight and fasting blood glucose concentrations were measured using weighing scale and Accu-Chek Active glucometer (Roche, Germany), respectively. After treatment for 3 weeks, the rats were fasted overnight for at least 16 h (4 p.m.–8 a.m.). The body weight and blood glucose concentrations were measured. The animals were then sacrificed under light diethyl ether anesthesia. Blood samples were collected in plain tubes and allowed to clot at room temperature. The blood samples were centrifuged at 1500 rpm for 10 min. The supernatants (sera) were collected and stored at −20 °C till further analysis.

### 2.6. Biochemical Analysis

The serum concentrations of total cholesterol (TC), triglycerides (TGs) and high density lipoprotein (HDL) cholesterol were determined using Agappe kits (Agappe Diagnostics, Knonauerstrasse, Cham, Switzerland) on EMP-168 Biochemical Analyzer according to the manufacturer’s instructions. Serum low density lipoprotein (LDL) and very low density lipoprotein (VLDL) cholesterol were estimated using the Friedewald equations [17].

LDL cholesterol = TC − [HDL cholesterol + (TG/5)]
(1)

VLDL cholesterol = TG/5
(2)

Non-HDL cholesterol was calculated by the formula:

Non-HDL cholesterol = TC − HDL cholesterol
(3)

### 2.7. Determination of Atherogenic Index (AI), Coronary Risk Index (CRI) and Cardiovascular Risk Index (CVRI)

The AI, CRI and CVRI were calculated using the formulae ([18,19]):

AI = LDL cholesterol/HDL cholesterol
(4)

CRI = TC/HDL cholesterol
(5)

CVRI = TGs/HDL cholesterol
(6)

### 2.8. Statistical Analysis

The results were analyzed using SPSS version 14. Data are expressed as mean ± SEM. Each group consisted of 6 rats (except the diabetic + 3.0 g/kg BW honey group which comprised 5 rats). Differences among treated groups were assessed by one way analysis of variance (ANOVA) followed by Tukey’s *post hoc* test. The results of initial and final blood glucose concentrations were analyzed using paired *t* test. *p* ≤ 0.05 was considered statistical significant.

## 3. Results

### 3.1. Effect of Honey on Percentage (%) Change in Body Weight (BW) of Diabetic Rats

**Figure 1:**
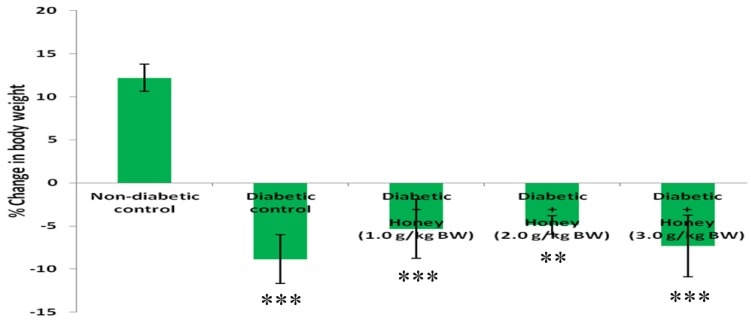
Effect of honey on % change in BW of diabetic rats. Data are expressed as mean ± SEM. ** & *** A significant decrease (*p* < 0.01 & *p* < 0.001) when compared with non-diabetic control.

The % change in BW was significantly reduced (*p* < 0.01 or *p* < 0.001) in diabetic rats including those treated with honey (Figure 1). Honey treatment did not improve % change in BW in diabetic rats.

### 3.2. Effects of Honey on Blood Glucose (BG) Concentrations and Percentage (%) Change in BG of Diabetic Rats

**Figure 2:**
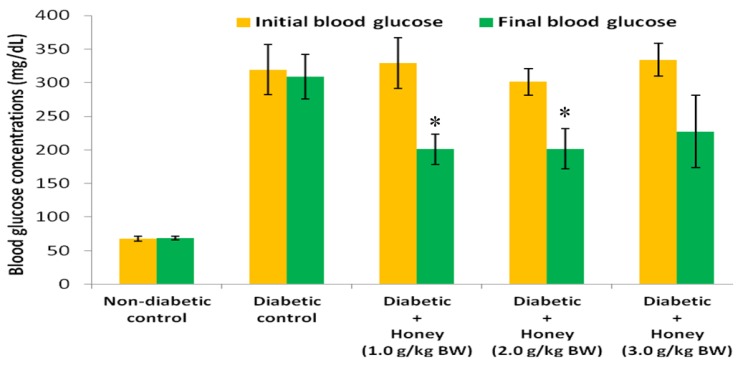
Effect of honey on BG concentrations of diabetic rats. Data are expressed as mean ± SEM. * A significant decrease (*p* < 0.05) when compared with initial BG concentrations within the same group.

**Figure 3:**
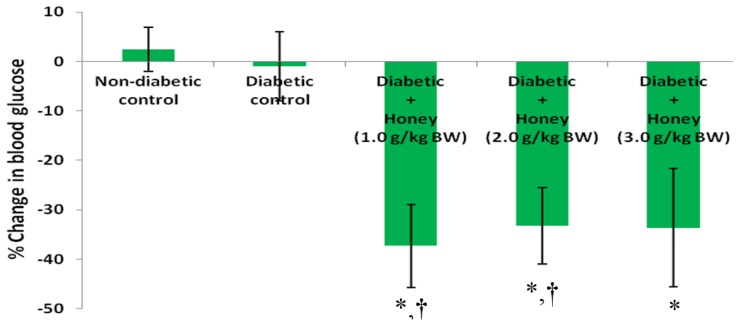
Effect of honey on % change in BG of diabetic rats. Data are expressed as mean ± SEM. * A significant decrease (*p* < 0.05) when compared with non-diabetic control; † A significant decrease (*p* < 0.05) when compared with diabetic control.

Figure 2 shows the effect of honey on BG concentrations of diabetic rats. The initial and final BG concentrations in non-diabetic and diabetic control groups did not differ. Compared with the initial BG concentrations, final BG levels were significantly (*p* < 0.05) lower in diabetic rats administered 1.0 or 2.0 g/kg BW of honey. Compared with diabetic control rats, only 1.0 or 2.0 g/kg BW of honey significantly (*p* < 0.05) reduced % change in BG in diabetic rats (Figure 3). The 3.0 g/kg BW of honey did decrease % change in BG but was not statistically significant.

### 3.3. Effects of Honey on Triglycerides (TG), High Density Lipoprotein (HDL), Non-HDL and Very Low Density Lipoprotein (VLDL) Cholesterol of Diabetic Rats

**Figure 4:**
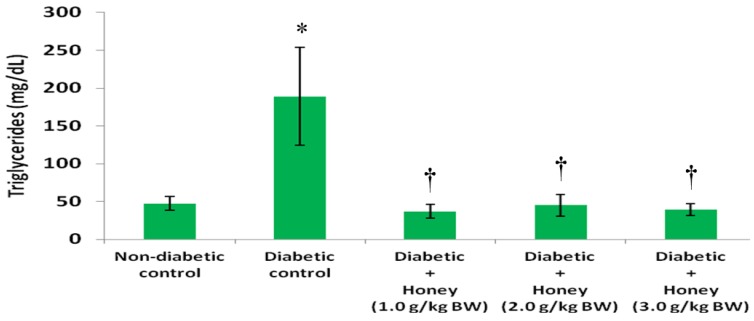
Effect of honey on triglycerides of diabetic rats. Data are expressed as mean ± SEM. * A significant increase (*p* < 0.05) when compared with non-diabetic control; † A significant decrease (*p* < 0.05) when compared with diabetic control.

**Figure 5:**
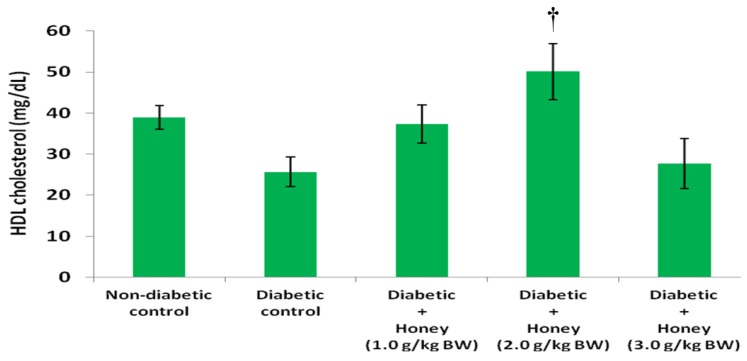
Effect of honey on HDL cholesterol of diabetic rats. Data are expressed as mean ± SEM. † A significant increase (*p* < 0.05) when compared with diabetic control.

**Figure 6:**
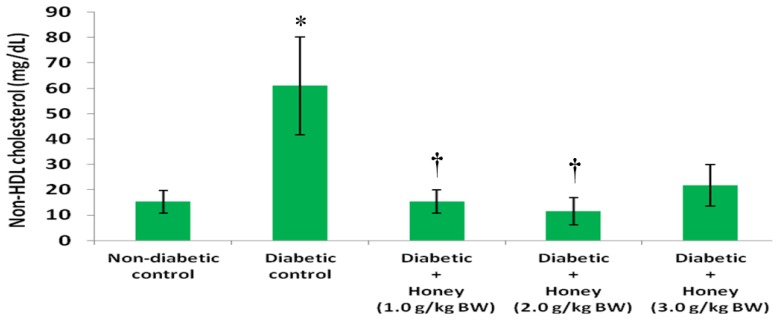
Effect of honey on non-HDL cholesterol of diabetic rats. Data are expressed as mean ± SEM. * A significant increase (*p* < 0.05) when compared with non-diabetic control; † A significant decrease (*p* < 0.05) when compared with diabetic control.

**Figure 7:**
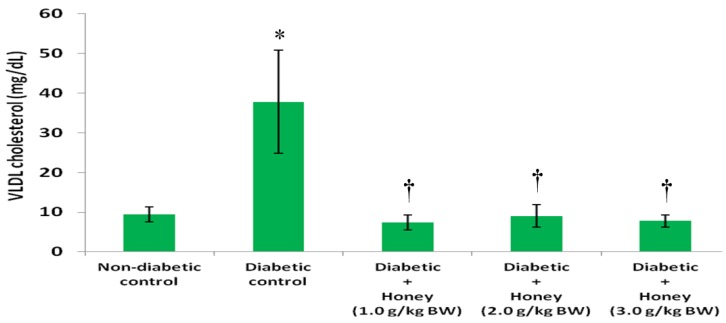
Effect of honey on VLDL cholesterol of diabetic rats. Data are expressed as mean ± SEM. * A significant increase (*p* < 0.05) when compared with non-diabetic control; † A significant decrease (*p* < 0.05) when compared with diabetic control.

The data on the effects of honey on TG, HDL, non-HDL and VLDL cholesterol in diabetic rats are presented in Figure 4, Figure 5, Figure 6 and Figure 7, respectively. The diabetic control group had significantly (*p* < 0.05) elevated levels of TG compared with non-diabetic rats. All the three doses of honey significantly (*p* < 0.05) reduced TG levels in diabetic rats (Figure 4). Though not statistically significant (*p* > 0.05), HDL cholesterol was reduced in diabetic control group (Figure 5). The 2.0 g/kg BW of honey significantly (*p* < 0.05) increased HDL cholesterol in diabetic rats. The diabetic control rats had significantly (*p* < 0.05) elevated levels of non-HDL cholesterol (Figure 6). Compared with diabetic control, the non-HDL cholesterol level was significantly (*p* < 0.05) reduced in diabetic rats administered 1.0 or 2.0 g/kg BW of honey. The reduction of non-HDL cholesterol produced by 3.0 g/kg BW of honey was not statistically significant. The diabetic control group had significantly (*p* < 0.05) elevated levels of VLDL cholesterol compared with non-diabetic rats (Figure 7). All the three doses of honey significantly (*p* < 0.05) reduced VLDL cholesterol levels in diabetic rats.

### 3.4. Effects of Honey on Coronary and Cardiovascular Risk Indices of Diabetic Rats

**Figure 8:**
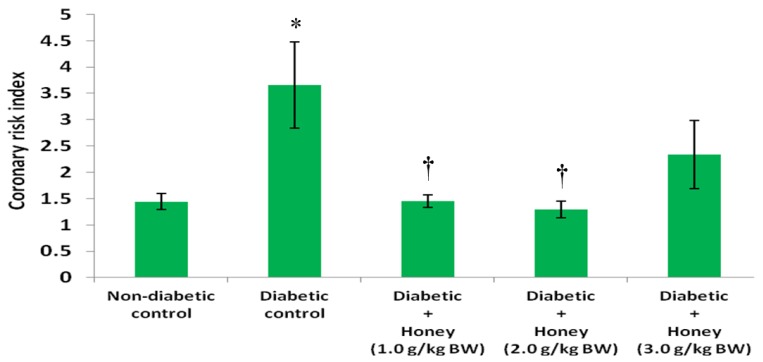
Effect of honey on coronary risk index of diabetic rats. Data are expressed as mean ± SEM. * A significant increase (*p* < 0.05) when compared with non-diabetic control; † A significant decrease (*p* < 0.05) when compared with diabetic control.

**Figure 9:**
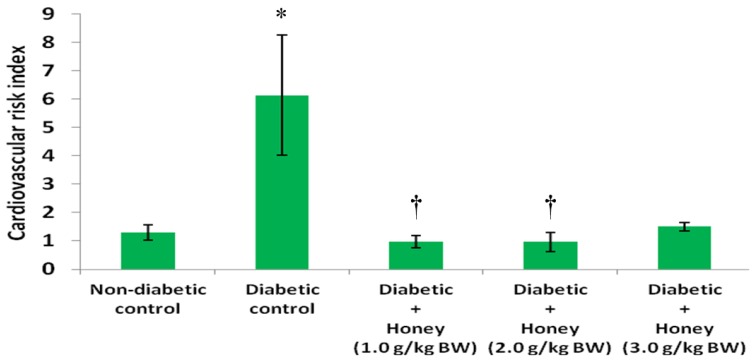
Effect of honey on cardiovascular risk index of diabetic rats. Data are expressed as mean ± SEM. * A significant increase (*p* < 0.05) when compared with non-diabetic control; † A significant decrease (*p* < 0.05) when compared with diabetic control.

Figure 8 shows the effect of honey on coronary risk index (CRI) in diabetic rats. The diabetic control rats had significantly (*p* < 0.05) higher levels of CRI compared with non-diabetic control. Administration of 1.0 or 2.0 g/kg BW of honey significantly (*p* < 0.05) reduced CRI in diabetic rats. The 3.0 g/kg BW of honey did reduce coronary risk index but not statistically significant. The effect of honey on cardiovascular risk index (CVRI) in diabetic rats is presented in Figure 9. The diabetic control rats had significantly (*p* < 0.05) higher CVRI compared with non-diabetic control. With the exception of 3.0 g/kg BW of honey, honey treatment significantly (*p* < 0.05) reduced CVRI in diabetic rats.

### 3.5. Effects of Honey on Total Cholesterol (TC), LDL Cholesterol and Atherogenic Index of Diabetic Rats

Table 1 shows the data on the effects of honey on TC, LDL cholesterol and atherogenic index. Though not statistically significant, the diabetic control rats had elevated levels of TC, LDL cholesterol and atherogenic index compared with non-diabetic rats. Although honey treatment (especially 1.0 or 2.0 g/kg BW of honey) reduced these parameters towards those of the non-diabetic rats, the reductions were not statistically significant (*p* > 0.05).

## 4. Discussion

In this study, a model of alloxan-induced diabetes was utilized to investigate the potential glucose lowering and hypolipidemic effect of Nigerian honey and also to evaluate if high doses of honey would deteriorate hyperglycemia and dyslipidemia. The three doses used in this study were selected based on previous findings as reported for Malaysian honey [7,20]. The lowest dose (1.0 g/kg BW) was shown to improve glycemic control and hyperlipidemia in streptozotocin-induced diabetic rats [21]. Two additional higher doses of honey (2.0 and 3.0 g/kg BW) were chosen as a follow-up to a study that reported exacerbating effect of honey on glycemic control in diabetic patients [13]. It was suggested that the administered high doses of honey might contribute to such deteriorating effect of honey [14].

The study found that alloxan-induced diabetic rats exhibited significant % reduction in body weight. Decreased body weight is commonly observed in alloxan-induced diabetic rodents [22]. This is attributed to break down of adipose tissue lipids and skeletal muscle protein [23]. Honey treatment did not improve % change in body weight in diabetic rats. This is in contrast with previous reports which found improved body weight following honey supplementation in streptozotocin-induced diabetic rats [7,20]. In the present study, honey treatment (1.0 or 2.0 g/kg BW) significantly reduced blood glucose levels in diabetic rats. These findings concur with previous results which demonstrated glucose lowering effect of honey in diabetic rats [7,20,22] and diabetic patients [24]. The potential mechanisms by which honey mediates its glucose lowering effect have been elaborated [25]. Fructose, oligosaccharides, antioxidants and mineral elements are some of the numerous honey constituents that may contribute to its glucose lowering effect [25,26,27]. Besides these individual constituents with glucose-lowering properties, their synergistic interactions will contribute considerably to glucose lowering effect of honey.

The results showed that, unlike 1.0 or 2.0 g/kg BW dose, 3.0 g/kg BW dose did not produce significant reduction in blood glucose concentrations. In a previous study, 0.2 g/kg BW of honey did not produce significant reduction in blood glucose level but 1.2 or 2.4 g/kg BW significantly decreased hyperglycemia [20]. As reported in that study, there was no additional benefit of doubling the dose of honey from 1.2 to 2.4 g/kg BW on hyperglycemia. Likewise in this study, there was no significant difference in the glucose lowering effect of 1.0 or 2.0 g/kg BW of honey. However, there is a clear disparity in the findings of these two studies. While the previous study revealed a dose-dependent glucose lowering response [20], this new study did not show that. In order to harmonize these inconsistencies, it is imperative to consider all the doses in these two studies in a context. The doses are 0.2, 1.0, 1.2, 2.0, 2.4 and 3.0 g/kg BW. While the 0.2 g/kg BW dose did not elicit glucose lowering effect most probably as a result of insufficient dose, the reason for the lack of significant glucose lowering response of 3.0 g/kg BW dose remains unknown. An analysis of these doses and their glycemic responses reveals a trend whereby honey at a particular dose (sub-therapeutic dose) did not exert glucose lowering effect. However, as the dose was increased, it produced glucose lowering effect. Additional dose increments also resulted in glucose lowering responses but with no additional glucose-lowering benefit. It then reached a dose at which further dose increment resulted in a loss of glucose lowering effect. Further studies are necessary to reveal if additional doses beyond 3.0 g/kg BW of honey will eventually deteriorate hyperglycemia.

Considering that 0.2 g/kg BW and 3.0 g/kg BW of honey did not elicit significant glucose lowering effect, based on existing studies, it can be inferred that the therapeutic doses of honey range between 1.0 and 2.4 g/kg BW. In view of the fact that any dose of honey selected between 1.0 and 2.4 g/kg BW will exert glucose lowering effect without further glucose-lowering benefit, it would be plausible to propose 1.0 g/kg BW as the optimal dose of honey. This dose (1.0 g/kg BW) of honey has been investigated in several other studies involving various diseases and therapeutic effects have been reported [28,29,30,31,32,33]. It is worth mentioning that even if 3.0 g/kg BW of honey had elicited considerable glucose lowering response, considering the lack of additional glucose-lowering response compared with 1.0 g/kg BW dose, it would still not be pharmacologically acceptable to utilize this dose or other higher doses for therapeutic purposes in diabetes studies especially clinical research. In view of the fact that 3.0 g/kg BW of honey neither reduced considerably nor increased blood glucose concentrations, it is uncertain if administration of this dose to diabetic rats over a longer period of time will alter hyperglycemic level. This could not be observed in this study, which was terminated at three weeks because of increased mortality in the diabetic control group. Another limitation is the fact that glycosylated hemoglobin (which was found to be increased in honey-supplemented diabetic patients [13]) was not measured in this study though it will not be valid.

Diabetic dyslipidemia constitutes an important modifiable risk factor for cardiovascular disorders. Consequently, the treatment of dyslipidemia is an important strategy in diabetes management [34]. The diabetic control rats had significantly elevated serum levels of TGs, non-HDL and VLDL cholesterol similar to what was reported previously [35]. The serum concentrations of TC and LDL cholesterol in diabetic rats were also increased but not statistically significant. In contrast, HDL cholesterol level was non-significantly lower in the diabetic control group than in the non-diabetic group. The findings on TC, LDL and HDL cholesterol in alloxan-induced diabetic control rats are comparable to those reported in a previous study [36]. Alloxan-induced diabetes is associated with reduced insulin level resulting from destruction of β-cells following alloxan administration [37]. This low level of insulin promotes hypertriglyceridemia and secretion of VLDL cholesterol [38,39]. Elevated levels of TGs in turn displace protein content of VLDL and LDL. This disproportionate imbalance of protein and triglyceride content of lipoproteins leads to decreased uptake of these lipoproteins by lipoprotein receptors [40]. The accumulation of these lipoproteins and TGs is implicated in many vascular disorders. LDL cholesterol is an independent risk factor for the development of coronary heart disease (CHD) [41]. The TGs, unlike LDL cholesterol, are not directly atherogenic, but it is an important risk factor for the development of cardiovascular disease (CVD) [25]. Administration of honey to diabetic rats markedly reduced TGs, non-HDL and VLDL cholesterol. However, honey (3.0 g/kg BW) did not significantly reduce non-HDL cholesterol. While the lowest dose of honey (1.0 g/kg BW) increased the HDL level towards that of the non-diabetic rats, the highest dose (3.0 g/kg BW) produced no such effect. In contrast, 2.0 g/kg BW of honey significantly increased HDL cholesterol. In previous studies, honey at a dose of 1.0 g/kg BW was found to significantly reduce TGs and VLDL cholesterol while it increased HDL cholesterol in diabetic rats [21,32]. Similar beneficial effects of honey on lipid abnormalities were also reported in both type 1 and type 2 diabetic patients [13,24,42]. The ameliorative effects of honey as observed in this study as well as those of the previous studies clearly demonstrate the benefits of honey in the treatment of dyslipidemia.

Even though honey supplementation of diabetic rats was associated with non-significant reduction of LDL cholesterol, it is noteworthy that honey administration significantly reduced elevated levels of both TGs and non-HDL cholesterol (which consists of the LDL, intermediate density lipoprotein (IDL) and VLDL cholesterol fractions). This is important because increased non-HDL cholesterol level together with hypertriglyceridemia in the presence of abnormal glucose metabolism increases risk of CVD [43]. Therefore, the marked ameliorative effects of honey on TGs and non-HDL cholesterol indicate honey can reduce risk of CVD. In this study, the effect of honey on lipid ratios (such as LDL/HDL cholesterol, TC/HDL cholesterol and TG/HDL cholesterol) in diabetic rats was evaluated. Assessment of lipid ratios is better than individual lipid parameters in predicting risk of atherogenicity, CHD and CVD [44]. Non-significant increase in LDL/HDL cholesterol was observed in diabetic control group. LDL/HDL cholesterol reflects atherogenic index (AI) [44]. Increased AI has also been reported in alloxan-induced diabetic rats [35]. Honey administration (especially 1.0 or 2.0 g/kg BW) tended to reduce atherogenic index. The data indicated TC/HDL cholesterol was markedly increased in diabetic control rats. The TC/HDL cholesterol is an index of CHD and is designated as coronary risk index (CRI) [45]. Honey supplementation (except the 3.0 g/kg BW dose) considerably reduced CRI which indicates honey can decrease the risk of CHD in diabetic rats. On the other hand, the TG/HDL cholesterol predicts the development of CVD and serves as cardiovascular risk index (CVRI) [46]. Recent evidence has also shown that TG/HDL cholesterol is an important predictor of cardiac disease mortality [47]. The results showed that the diabetic control rats had significantly elevated CVRI. However, some researchers reported non-significant elevation of this lipid ratio in alloxan-induced diabetic control mice [48]. Honey therapy (except the 3.0 g/kg BW dose) significantly reduced CVRI in diabetic rats. This finding therefore suggests that honey is capable of reducing risk of CVD in diabetic rats. Increased TG/HDL-C ratio is also a reflection of elevated levels of small and dense subclass of LDL cholesterol (sdLDL), which contribute to increased cardiovascular risk [49]. Hence, the decreased TG/HDL cholesterol in honey-treated diabetic rats suggests that honey supplementation reduced sdLDL in diabetic rats.

In view of the role of insulin in lipid metabolism and prevention of hypertriglyceridemia [50], it is plausible to propose a role of insulin in the hypolipidemic effect of honey. Based on the current literature, honey may ameliorate dyslipidemia in part via enhanced release of insulin from the remnant pancreatic β-cells. This proposition is supported by compelling evidence from previous studies. Some beneficial effects of honey on insulin have been reported in human subjects [42,51]. Similarly, honey has been shown to increase serum insulin level in streptozotocin-induced diabetic rats [21] and C-peptide (a peptide released from the β-cells during cleavage of insulin from proinsulin) in diabetic patients [24]. Histological examination of pancreata from honey-treated diabetic rats has also revealed less severe injury, incomplete restoration of cellular population as well as bigger size of the islets of Langerhans compared with pancreata from untreated diabetic rats [52]. Besides, honey has been shown to protect the pancreas against oxidative stress [53]. This may, in turn, contribute to protection of β-cells against hyperglycemia-induced oxidative damage [52,53,54]. All these pancreatic protective effects of honey will help to preserve the β-cells which invariably will contribute to increased serum insulin levels as reported [21]. Increased secretion of insulin will enhance lipogenesis and inhibit lipolysis leading to amelioration of hyperlipidemia [55].

It is worthy of note that, compared to the other two doses, the 3.0 g/kg BW of honey ameliorated dyslipidemia to a less extent or partially. This is evident by the lack of significant effect of this dose on serum levels of HDL, non-HDL cholesterol, TC/HDL cholesterol and TG/HDL cholesterol. Furthermore, an analysis of the data presented in Table 1 reveals the values of LDL cholesterol and atherogenic risk index in the diabetic rats treated with 3.0 g/kg BW of honey were elevated towards those of the diabetic control rats. These data on the lipid parameters and lipid ratios seem to suggest that honey at 3.0 g/kg BW lost its hypolipidemic effect. Interestingly, this reduced or loss of hypolipidemic effect of 3.0 g/kg BW of honey is in agreement with its loss of glucose lowering effect. Additional studies involving higher doses or the same dose but for a longer duration of treatment may help to reveal if honey can worsen dyslipidemia.

## 5. Conclusions

This study shows that, using comparable doses as reported for Malaysian honey, Nigerian honey ameliorates hyperglycemia and dyslipidemia in alloxan-induced diabetic rats. Thus, this study adds to the limited available evidence that the glucose lowering and hypolipidemic effects of honey are not restricted to honey samples of a particular geographical origin. In addition, the study extends previous findings by reporting that the beneficial effects of honey on glucose and lipid metabolism as well as lipid ratios may be lost at high doses of honey. The study, however, did not find any deteriorating effect of the highest dose of honey (3.0 g/kg BW) on hyperglycemia and dyslipidemia. Considering that the therapeutic benefits of honey on metabolic derangements tended to be lost or reduced at the highest dose, it remains unclear if 3.0 g/kg BW of honey or higher doses administered over a longer duration of time would worsen hyperglycemia and dyslipidemia in diabetes.

## Figures and Tables

**Table 1:** Effects of honey on total cholesterol (TC), low density lipoprotein (LDL) cholesterol and atherogenic index.

Group	TC (mg/dL)	LDL Cholesterol (mg/dL)	Atherogenic Index
Non-diabetic control	54.3 ± 2.4	8.0 ± 3.7	0.2 ± 0.1
Diabetic control	86.7 ± 18.1	23.2 ± 13.0	1.1 ± 0.7
Diabetic + Honey (1.0 g/kg BW)	52.6 ± 5.4	7.9 ± 2.9	0.2 ± 0.1
Diabetic + Honey (2.0 g/kg BW)	61.6 ± 5.6	4.4 ± 2.8	0.1 ± 0.1
Diabetic + Honey (3.0 g/kg BW)	49.4 ± 3.0	21.6 ± 7.5	1.0 ± 0.6

Data are expressed as mean ± SEM.

## Abbreviations

The following abbreviations are used in this manuscript:

CVD: cardiovascular disease
CHD: coronary heart disease
NAFDAC: National Agency for Food and Drug Administration Control
BW: body weight
BG: blood glucose
TC: total cholesterol
TGs: triglycerides
LDL: low density lipoprotein
VLDL: very low density lipoprotein
HDL: high density lipoprotein
Non-HDL: non-high density lipoprotein
AI: atherogenic index
CRI: coronary risk index
CVRI: cardiovascular risk index
SEM: standard error of mean
ANOVA: analysis of variance
